# Infection and Vaccine Induced Spike Antibody Responses Against SARS-CoV-2 Variants of Concern in COVID-19-Naïve Children and Adults

**DOI:** 10.1007/s10875-023-01540-5

**Published:** 2023-07-05

**Authors:** Aleha Pillay, Avani Yeola, Fiona Tea, Martina Denkova, Samuel Houston, Rebecca Burrell, Vera Merheb, Fiona X. Z. Lee, Joseph A. Lopez, Lilly Moran, Ajay Jadhav, Katrina Sterling, Catherine L. Lai, Tennille L. Vitagliano, Anupriya Aggarwal, Dan Catchpoole, Nicholas Wood, Tri Giang Phan, Ralph Nanan, Peter Hsu, Stuart G. Turville, Philip N. Britton, Fabienne Brilot

**Affiliations:** 1 Brain Autoimmunity Group, Kids Neuroscience Centre, Kids Research at the Children’s Hospital at Westmead, Sydney, New South Wales Australia; 2 School of Medical Sciences, Faculty of Medicine and Health, The University of Sydney, Sydney, New South Wales Australia; 3 Kids Research at the Children’s Hospital at Westmead, Sydney, New South Wales Australia; 4 National Center for Immunisation Research and Surveillance, the Sydney Children’s Hospitals Network, Sydney, New South Wales Australia; 5 The Kirby Institute, The University of New South Wales, Sydney, New South Wales Australia; 6 Garvan Institute of Medical Research, Sydney, New South Wales Australia; 7 St Vincent’s Healthcare Clinical Campus, School of Clinical Medicine, Faculty of Medicine and Health, The University of New South Wales, Sydney, New South Wales Australia; 8 Charles Perkins Center and Sydney Medical School Nepean, Faculty of Medicine and Health, The University of Sydney, Sydney, New South Wales Australia; 9 Department of Allergy and Immunology, The Children’s Hospital at Westmead, Sydney, New South Wales Australia; 10 Sydney Institute for Infectious Disease, Faculty of Medicine and Health, The University of Sydney, Sydney, New South Wales Australia; 11 Brain and Mind Centre, The University of Sydney, Sydney, New South Wales Australia

**Keywords:** Pediatric COVID-19, adult COVID-19, SARS-CoV-2, variant of concern, epistasis, vaccine, Spike antibody, cross-reactivity

## Abstract

Although a more efficient adaptive humoral immune response has been proposed to underlie the usually favorable outcome of pediatric COVID-19, the breadth of viral and vaccine cross-reactivity toward the ever-mutating Spike protein among variants of concern (VOCs) has not yet been compared between children and adults. We assessed antibodies to conformational Spike in COVID-19-naïve children and adults vaccinated by BNT162b2 and ChAdOx1, and naturally infected with SARS-CoV-2 Early Clade, Delta, and Omicron. Sera were analyzed against Spike including naturally occurring VOCs Alpha, Beta, Gamma, Delta, and Omicron BA.1, BA.2, BA.5, BQ.1.1, BA2.75.2, and XBB.1, and variants of interest Epsilon, Kappa, Eta, D.2, and artificial mutant Spikes. There was no notable difference between breadth and longevity of antibody against VOCs in children and adults. Vaccinated individuals displayed similar immunoreactivity profiles across variants compared with naturally infected individuals. Delta-infected patients had an enhanced cross-reactivity toward Delta and earlier VOCs compared to patients infected by Early Clade SARS-CoV-2. Although Omicron BA.1, BA.2, BA.5, BQ.1.1, BA2.75.2, and XBB.1 antibody titers were generated after Omicron infection, cross-reactive binding against Omicron subvariants was reduced across all infection, immunization, and age groups. Some mutations, such as 498R and 501Y, epistatically combined to enhance cross-reactive binding, but could not fully compensate for antibody-evasive mutations within the Omicron subvariants tested. Our results reveal important molecular features central to the generation of high antibody titers and broad immunoreactivity that should be considered in future vaccine design and global serosurveillance in the context of limited vaccine boosters available to the pediatric population.

**Supplementary Information:**

The online version contains supplementary material available at 10.1007/s10875-023-01540-5.

## Introduction

In children, the severity of severe acute respiratory syndrome coronavirus 2 (SARS-CoV-2) infection is attenuated, adverse disease outcome is less frequent, and lower rates of pediatric hospitalizations have been reported [1–3]. Furthermore, SARS-CoV-2 vaccination recommendations have been delayed in children, and once implemented, still differ from adults [4]. In many countries, vaccination rates and number of boosters remain higher in adults than in children, and with emerging viral variants, the risk of breakthrough infection is still highly relevant for children [5]. These differences may be underlined by distinct innate and adaptive immunity in children and adults upon SARS-CoV-2 infection [6, 7], and recent data has suggested that a memory response in children may not be as robust as in adults [8]. Studies on the humoral response have also been varied with reports of a more robust and sustained antibody response in children [9, 10], whereas similar or lower antibody titers were also documented [11].

SARS-CoV-2 Spike glycoprotein is fundamental to viral tropism and infectivity. Spike, especially its receptor binding domain (RBD), is also the immunodominant antigenic region of protective neutralizing antibodies generated post-infection [12–16] and vaccination [17]. Monitoring of antibody titers remains the most useful approach to assess vaccine effectiveness, immune responsiveness, and viral protection [18]. Antibodies generated by vaccination would ideally be protective against the dominant global variant and emerging viral variants, as defined by the World Health Organization (WHO) and based on viral characteristics of immune evasion, viral infectivity, and transmissibility [19]. These characteristics differ across variants of concern (VOCs), and are attributed predominantly to specific mutations across Spike [20–23] that lead to loss of binding by therapeutic monoclonal antibodies, especially to the new variants such as BQ.1.1, BA2.75.2, and XBB.1 [24–28]. Indeed, immune evasiveness has been observed after infection with Omicron BA.1, BA.2, BA.5, BQ.1.1, and XBB.1 subvariants in adults [27, 28]. Although lower Spike antibody titers have been detected in Omicron-infected children compared to adults [11], comparative data on SARS-CoV-2 humoral cross-reactive binding against variants, such as BQ.1.1, BA2.75.2, and XBB.1 between children and adults, is currently scarce. Examining the antibody responses toward viral variants across the lifespan can resolve the deleterious effect of mutations, as well as provide data on epistasis, by which mutations at some sites modulate the effects of others.

Herein, the current study utilizes molecular cloning and sensitive antibody detection by flow cytometry to delineate the contribution of the Spike RBD in vaccinated children and adults and those naturally infected by SARS-CoV-2 variants. This will guide ongoing variant-proof vaccine development strategies to select optimal regions that should be targeted to induce potent viral neutralizing antibody responses.

## Methods

### Study Cohort

**Fig. 1:**
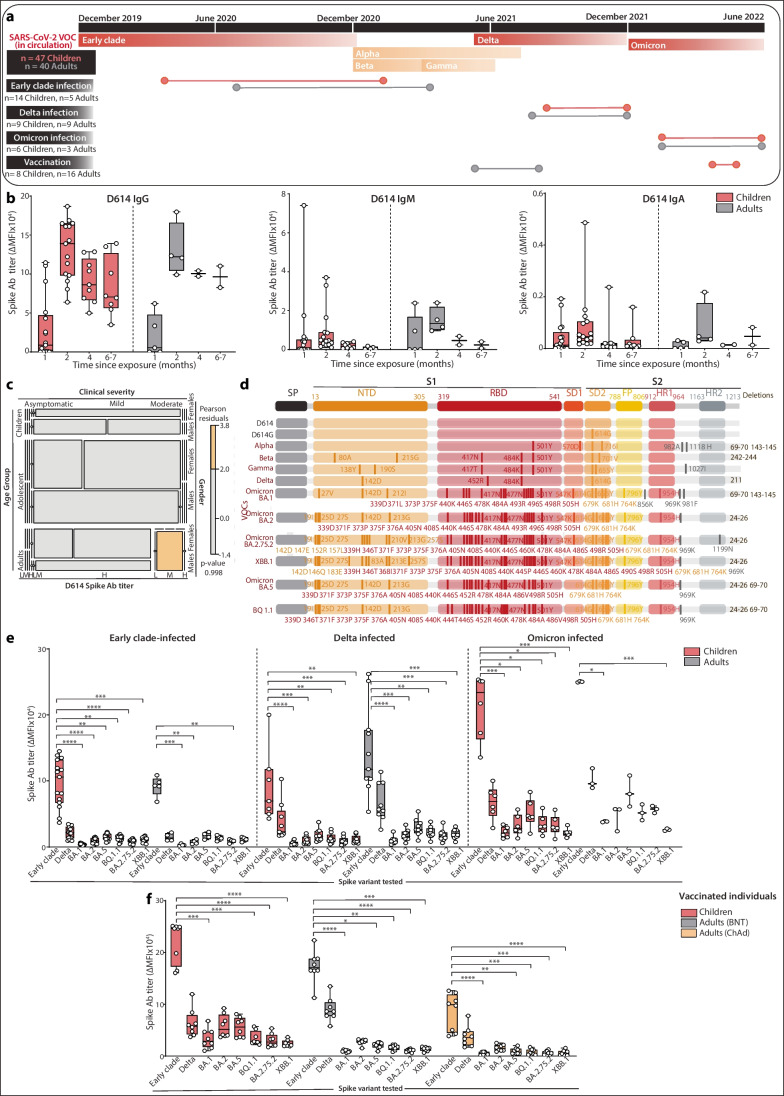
Persistence of sera Spike antibodies against natural variants in children and adults. **a** Schematic showing timeline of different waves of infections in Australia. Convalescent patient sera from individuals naturally infected with Early Clade, Delta, or Omicron Spike or vaccinated individuals were examined for SARS-CoV-2 antibodies in a total of 47 children and 40 adults. In each cohort, the dots indicate the beginning and end of the sample collection timeframe. **b** IgG, IgM, and IgA responses against Spike over time in Early Clade–infected children (ages 0–18 years old, peach) and adults (>18 years old, gray) shown since viral exposure. **c** COVID-19 severity caused by the Early Clade SARS-CoV-2 was mostly mild or asymptomatic in children, whereas “close contact” inter-related adults presented more often with a moderate disease which was associated with higher Spike IgG levels. **d** Schematic indicating primary mutations in VOCs within the Spike protein. **e** Peak IgG responses generated against Early clade, Delta, BA.1, BA.2, BA.5, BQ.1.1, BA2.75.2, and XBB.1 Spike VOCs in naturally infected individuals. **f** Peak IgG responses generated against Early Clade, Delta, BA.1, BA.2, BA.5, BQ.1.1, BA2.75.2, and XBB.1 Spike VOCs in vaccinated individuals. All points shown were reported seropositive against a control cohort and calculated positive threshold. ∆MFI, delta median fluorescence intensity; Ab, antibody; VOIs, variants of interest; VOCs, variants of concern. *p* values were determined by comparing VOC Spikes vs Early Clade Spike. *p<0.05, **p<0.01, ***p<0.001, ****p<0.0001

SARS-CoV-2 antibody response against Spike variants was characterized in 87 COVID-19-naïve children (*n*=47) and adults (*n*=40). Child and adult samples were collected when individuals were COVID-19-naïve up to the second wave of COVID-19 in Sydney which occurred after a period of a time when there were no SARS-CoV-2 cases in the community [29]. PCR positivity was reported in patients naturally infected with Early Clade (*n*=14 children, *n*=5 adults), Delta (*n*=9 children, *n*=9 adults), and Omicron (*n*=6 children, *n*=3 adults) (Table 1; Fig. 1a). For some patients, clinical respiratory samples were sequenced according to previously described methods [12, 30] and uploaded to GISAID (www.gisaid.org). Sera from 24 individuals vaccinated with BNT162b2 (*n*=8 children and *n*=8 adults) and ChAdOx1 (*n*=8 adults) were also collected for antibody analyses. Sera in vaccinated patients were collected within 35 days following their second vaccine dose (Table 1). All vaccinated children and adults were COVID-19-naïve and had not been infected by SARS-CoV-2 before vaccination as measured by seronegativity for Spike immunoglobulin G. Forty-eight age-matched healthy and non-inflammatory disorder pre-pandemic control sera (*n*=24 children, *n*=24 adults) were used to determine patient seropositivity against Spike variants (Table 1).

**Table 1:** Demographics of infected and vaccinated children and adults

		Patients, *n*	Serial samples, *n*	D614 IgG positivity, *n positive (%)*	Age at exposure date in seropositive individuals, *(median years, IQR)*	Sex, *F:M*	Time between exposure and date of first collected sample (median days, IQR)	COVID-19 clinical severity in seropositive individuals (*n*)	SARS-CoV-2 variant at diagnosis (*n*, %)	Patient sample collected at the peak immune respons e, *n*	Age at peak sample after infection/vaccination *(median years, IQR)*	Sex, *F:M*	Time between exposure and collection date of peak sample/second vaccination sample *(median days, IQR)*
Naturally infected individuals	Early Clade–infected	21	67	18 (86)	15 (13–16)	11:10	11 (9–14)	Asymptomatic (0)	D614/D614G (9/18, 50%)	18	15 (13–16)	5:4	38 (38–40)
								Mild (17)					
								Moderate (1)					
								Severe (0)					
	Children (0–18)	16	53	14 (88)	15 (11–15)	1:1	12 (9–15)	Asymptomatic (0)		14	15 (13–15)	1:1	32 (32–41)
								Mild (14)					
								Moderate (0)					
								Severe (0)					
	Adults (>18)	5	14	5 (100)	42 (34–58)	3:2	11 (10–11)	Asymptomatic (0)		5	43 (35–58)	3:2	30 (30–35)
								Mild (3)					
								Moderate (1)					
								Severe (0)					
	Delta-infected	26	44	18 (69)	15 (9–34)	7:6	40 (27–42)	Asymptomatic (2)	Delta (18/18, 100%)	18	21 (10–34)	7:11	40 (35–42)
								Mild (9)					
								Moderate (2)^a^					
								Severe (5)^b^					
	Children	15	20	9 (60)	9 (4–12)	1:2	40 (37–55)	Asymptomatic (0)		9	9 (4–14)	4:5	40 (34–40)
								Mild (6)					
								Moderate (1)^c^					
								Severe (2)^c^					
	Adults	11	24	9 (82)	34 (31–38)	9:2	38 (15–41)	Asymptomatic (2)		9	34 (30–36)	7:2	42 (40–101)
								Mild (3)					
								Moderate (1)^c^					
								Severe (3)^d^					
	Omicron-infected	16	36	9 (56)	27 (13–43)	1:1	11 (9–12)	Asymptomatic (1)	BA.1 (2/9, 22%) BA.2 (7/9, 78%)	9	14 (10–41)	4:5	38 (29–46)
								Mild (14)					
								Moderate (1)					
								Severe					
	Children	8	19	6 (75)	12 (10–14)	1:3	11 (9–12)	Asymptomatic (1)		6	12 (10–14)	1:2	34 (21–40)
								Mild (7)					
								Moderate					
								Severe					
	Adults	8	17	3 (38)	44 (41–48)	3:1	11 (9–21)	Asymptomatic		3	46 (44–47)	2:1	46 (42–46)
								Mild (7)					
								Moderate (1)					
								Severe					
	International WHO standard	11e	-	1 (100)	-	-	-		D614/D614G				
Vaccinated individuals	Vaccinated adults	16	-	100		3:1	N/A	N/A		16	33 (26–39)	-	35 (31–36)
	BNT162b2	8	-	100		7:1	N/A	N/A		8	32 (26–39)	7:1	34 (31–36)
	ChAdOx1	8	-	100		5:3	N/A	N/A		8	33 (27–39)	5:3	35 (33–36)
	Vaccinated Children (BNT162b2)	8	-	100		1:3	N/A	N/A		8	10 (6–11)	1:3	32 (30–33)
Pre-pandemic controls	Children	24	-	0 (0)	X		-						
	Adults	24	-	0 (0)	X age + range for the cohort		-						

^a^Two patients required respiratory support

^b^Three patients admitted to ICU, four patients required respiratory support

^c^One patient required respiratory support

^d^All patients admitted to ICU and required respiratory support

^e^ WHO International National Institute for Biological Standards and Control standards made from pooled convalescent patient serum

### Ethics

This study was conducted in accordance with the Declaration of Helsinki. The Institutional Review Board from the Sydney Children’s Hospital Network Human Research Ethics Committee reviewed and approved the overall studies (2020/ETH00837, 2021/ETH11346, Sydney Australia). Written consent was obtained from all participants or their parents or legal guardians.

### Detection of Spike Antibody by Live Flow Cytometry Cell-Based Assay

**Fig. 2:**
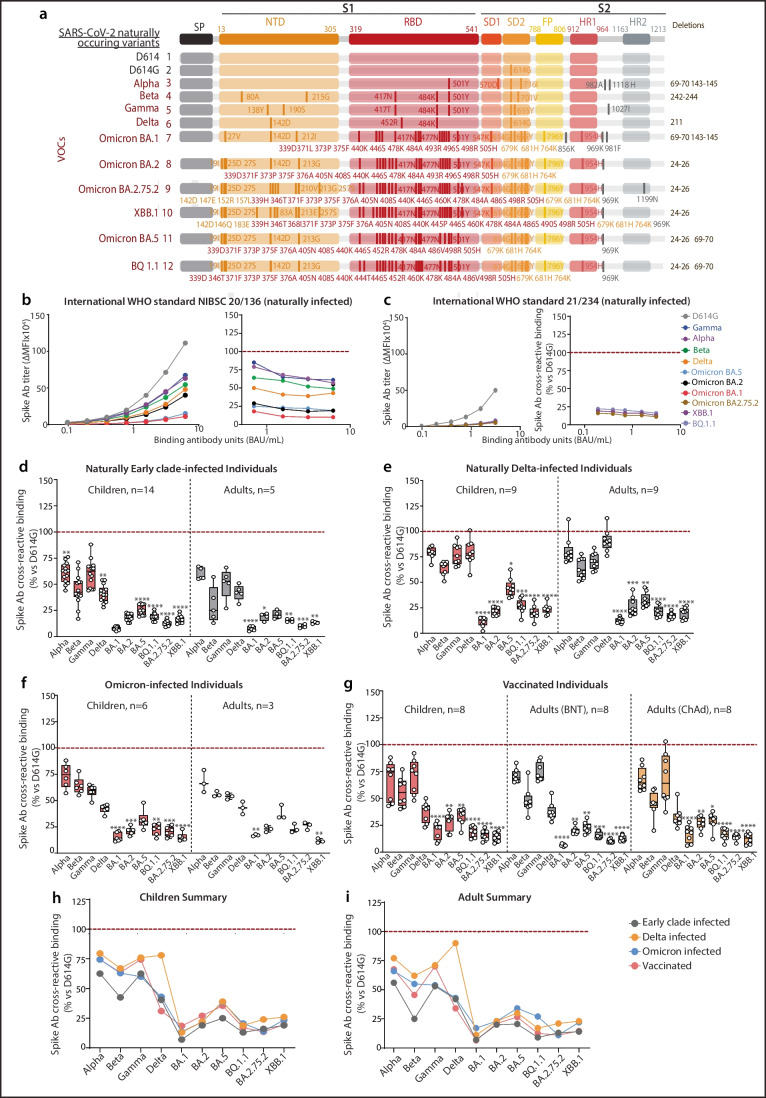
Spike IgG immunoreactivity against VOCs in naturally infected and vaccinated children and adults. **a** Schematic showing mutations within VOCs along SARS-CoV-2 Spike protein. **b** WHO International NIBSC SARS-CoV-2 standard (20/136) tested against VOCs and titer of IgG against VOCs. **c** WHO International NIBSC SARS-CoV-2 standard (21/234) tested against VOCs and cross-reactive binding compared to Early Clade Spike (percentage). Evaluation of Spike VOCs cross-reactive binding in comparison to Early Clade Spike in sera samples from **d** naturally Early Clade–infected children (*n*=14) and adults (*n*=5), **e** naturally Delta-infected children (*n*=9) and adults (*n*=9), **f** naturally Omicron-infected children (*n*=6) and adults (*n*=3), and **g** vaccinated children (*n*=8), adults who received BNT162b2 vaccine (*n*=8), and adults who received ChAdOx1 vaccine (*n*=8). Summarized comparison of immunoreactivity across VOCs in different cohorts of **h** children and **i** adults. All points shown were collected at the peak of the immune response (Table 1) and were seropositive against a control cohort and calculated positive threshold. The dotted line shows the reference binding on Early Clade Spike. *p* values were determined by comparing VOC Spikes vs Early Clade Spike. *p<0.05, **p<0.01, ***p<0.001, ****p<0.0001. ∆MFI, delta median fluorescence intensity; Ab, antibody; VOCs, variants of concern

**Fig. 3:**
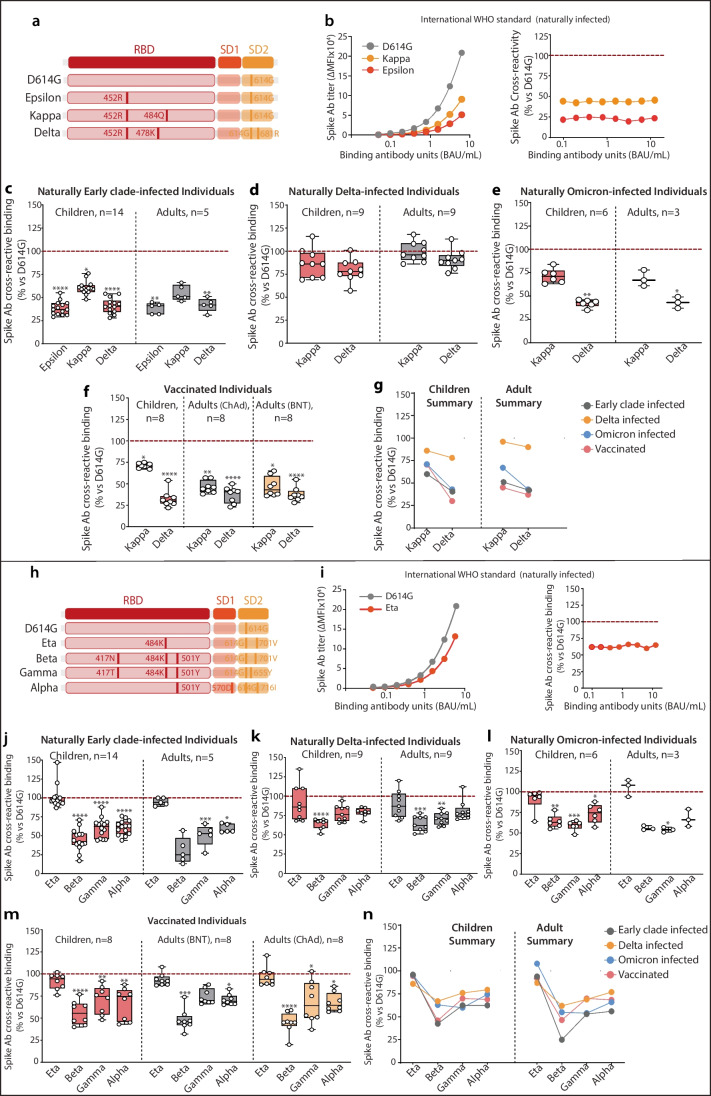
Additive effects of mutations within SARS-CoV-2 Spike RBD. **a** Schematic showing mutations in different VOCs at specific positions within the SARS-CoV-2 Spike protein. **b** WHO International NIBSC SARS-CoV-2 standard tested on variants Delta, Kappa, and Epsilon, titer of Ab binding to variants (left) and cross-reactive binding compared to Early Clade Spike (percentage) (right). Proportion of Spike VOC cross-reactive binding compared to Early Clade Spike in sera samples from **c** Early Clade–infected individuals, **d** Delta-infected individuals, **e** Omicron-infected individuals, and **f** vaccinated individuals. **g** Summarized comparison of immunoreactivity toward Kappa and Delta Spike variants across different cohorts. **h** Mutations within SARS-CoV-2 Spike variants within the RBD. **i** WHO International NIBSC SARS-CoV-2 standard tested on Eta and Early Clade Spike variants, titers of Ab binding to variants (left) and cross-reactive binding compared to Early Clade Spike (right). Cross-reactive binding compared to Early Clade Spike VOC against Eta, Beta, Gamma, and Alpha Spike variants in **j** Early Clade–infected individuals, **k** Delta-infected individuals, **l** Omicron-infected individuals, and **m** vaccinated individuals. **n** Summarized comparison of cross-reactive binding toward Eta, Beta, Gamma, and Alpha Spike variants across different cohorts. All points shown were collected at the peak of the immune response (Table 1) and were seropositive against a control cohort and calculated positive threshold. The dotted line shows the reference binding on Early Clade Spike. *p* values were determined by comparing VOC Spikes vs Early Clade Spike. *p<0.05, **p<0.01, ***p<0.001, ****p<0.0001. ∆MFI, delta median fluorescence intensity; Ab, antibody; VOCs, variants of concern.

**Fig. 4:**
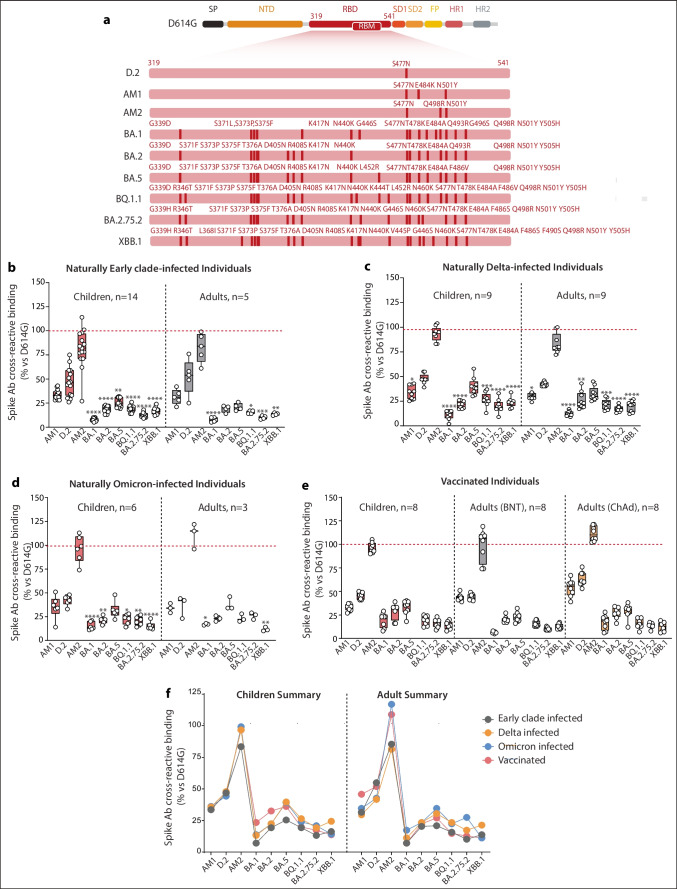
Epistatic effect of mutations within the Spike RBD. **a** Schematic of mutation within the RBD for AM1 Spike, D.2 Spike, AM2 Spike, and Omicron subvariants. **b** Cross-reactive binding against AM1, D.2, AM2, BA.1, BA.2, BA.5, BQ.1.1, BA2.75.2, and XBB.1 Spikes in sera from Early Clade–infected individuals, **c** Delta-infected individuals, **d** Omicron-infected individuals, and **e** vaccinated individuals. **f** Summarized comparison of cross-reactive binding toward AM1 Spike, D.2 Spike, and AM2 Spike across different cohorts. All points shown were collected at the peak of the immune response (Table 1) and were seropositive against a control cohort and calculated positive threshold. The dotted line shows the reference binding on Early Clade Spike. *p* values were determined by comparing VOC Spikes vs Early Clade Spike. *p<0.05, **p<0.01, ***p<0.001, ****p<0.0001. ∆MFI, delta median fluorescence intensity; Ab, antibody; VOCs, variants of concern

Detection and quantification of Spike antibodies in patient sera were performed as previously described (Supplementary Methods) [12, 31–33]. Antibody assessment by flow cytometry is quantitative, highly sensitive, and specific, and used for clinical purposes of neuroimmunological disorder diagnosis [32, 34–36]. Full-length conformational Spikes, generated as previously described [12], included SARS-CoV-2 VOCs Early Clade D614 and D614G, Alpha, Beta, Delta, Gamma, and Omicron BA.1, BA.2, BA.5, BQ.1.1, BA2.75.2, and XBB.1, and SARS-CoV-2 variants of interest (VOIs) D.2, Kappa, Epsilon, Eta, and two artificial Spikes (AM1 and AM2 Spikes) (Supplementary Table S1). Median fluorescence intensity (MFI) by flow cytometry has been reported to be a strong correlate of antibody titer and live virus neutralization capacity against Early Clade, Delta, and Omicron BA.1, BA.2, BA.5, BQ.1.1, and XBB.1 SARS-CoV-2 (Supplementary Methods and Supplementary Fig. S1). A minimum of 30,000 ΔMFI is sufficient to neutralize Early Clade SARS-CoV-2 [12, 26, 28, 37, 38]. To investigate the cross-reactive binding to emerging VOIs and VOCs, sera collected at peak response (within 2 months post SARS-CoV-2 exposure) were analyzed. Antibody cross-reactive binding toward Spike variants as shown in Figs. 2, 3, and 4 was expressed as a percentage of binding compared to the reference Early Clade Spike (D614G). A median binding of 75–100% was described as strong cross-reactive binding to the Spike of interest (vs Early Clade Spike); 50–75% indicated moderate binding, 25–50% indicated weak binding, and 0–25% indicated limited antibody binding. The WHO International National Institute for Biological Standards and Control (NIBSC) standards [39, 40] (20/136 and 21/234) made from pooled convalescent plasma from recovered Early Clade SARS-CoV-2 patients were assessed alongside patient sera and used as internal references of defined concentration (BAU/mL) in the flow cytometry assays [41].

### Statistics

Mosaic plots were performed in RStudio (version 4.2), and Pearson residuals were plotted. The nonparametric Kruskal-Wallis test followed by Dunn’s multiple comparison test was performed and adjusted *p* values were shown in figures and text when significant (Supplementary Methods). Comparisons between Spike antibodies (titers and cross-reactive binding) in children and adults were performed using the Mann-Whitney *U*-test and Bonferroni correction for multiple comparison tests (Supplementary Table S2). Based on a reported 42% difference in antibody titers [42], a sample size of Early Clade–infected *n*=14 children and *n*=5 adults provided a statistical power of 99.9% (Supplementary Methods).

## Results

### VOC-Specific Assessment of Spike Antibody Reveals Risk of Titer Overestimation in Delta- and Omicron-Infected Children and Adults

We first determined seropositivity against Early Clade Spike in 67 sera samples from 21 Early Clade–infected inter-related (*n*=16 children, *n*=5 adults; Fig. 1a). All PCR-negative children and adults were seronegative, and PCR-positive patients with a minimum of 10 days since viral exposure were seropositive for antibodies against Spike (Table 1). The time since virus exposure across all samples was similar between children and adults, and severity of COVID-19 was mostly mild or asymptomatic (Table 1). IgG, IgM, and IgA antibody titers generated 1 to 6 months following viral exposure were similar between children and adults (Fig. 1b, ns). Of note, child IgG responses were maintained at similar levels as adults over time, up to 196 and 209 days post exposure (Fig. 1b). IgG, IgM, and IgA against Early Clade SARS-CoV-2, the viral variant that had infected these individuals, peaked at a median of 32 (IQR 32–41) and 30 days (IQR 30–35) after viral exposure in children and adults, respectively (Fig. 1b), and across all Spikes analyzed (Supplementary Fig. S2). Early Clade Spike IgG antibody titers persisted for an extended period, with no patient seroreverting for Early Clade Spike IgG or IgA, even after 7 months following viral exposure. One adult and six children became negative for Spike IgM at an average of 163 days after viral exposure. As expected, COVID-19 caused by Early Clade SARS-CoV-2 was mostly mild or asymptomatic in children, and most “close contact” adults from the same households were also affected by mild COVID-19 (Table 1). Higher Spike IgG titers were associated with the few adults with moderate disease and consistent with previous observations reporting an association between antibody titers and COVID-19 severity (Fig. 1c).

We then examined the levels of Spike antibodies generated at the immune response peak after natural infection and immunization in cohorts of COVID-19-naïve individuals up to 46 days post exposure (Table 1; Fig. 1a). Sera collected at peak response from 19 Early Clade–infected individuals (*n*=14 children, *n*=5 adults); 18 Delta-infected individuals (*n*=9 children, *n*=9 adults), nine Omicron-infected individuals (*n*=6 children, *n*=3 adults), and 24 never infected vaccinated individuals (*n*=8 children and *n*=8 adults with BNT162b2 vaccine, and *n*=8 adults with ChAdOx1 vaccine) (Table 1) were assessed for SARS-CoV-2 antibody against Early Clade, Delta, BA.1, BA.2, BA.5, BQ.1.1, BA2.75.2, and XBB.1 Spikes (Fig. 1d). SARS-CoV-2 infection was confirmed by sequencing, and by the prevalence of a particular variant at the time of exposure (Table 1; Fig. 1a) [43, 44]. SARS-CoV-2 antibody titers against Early Clade, Delta, BA.1, BA.2, BA.5, BQ.1.1, and XBB.1 Spikes were well correlated with RBD-targeting live virus neutralization of these variants (Supplementary Fig. S1).

Early Clade Spike IgG antibody titers after Early Clade infection were slightly higher in children than in adults although not statistically significant (Fig. 1e; Supplementary Table S2, ns). Absolute levels of Spike IgG antibody significantly decreased when the sera from children were tested against Spike from VOCs, such as BA.1, BA.2, BA.5, BQ.1.1, BA2.75.2, and XBB.1, compared to Early Clade Spike (Fig. 1e). In adults, this effect was also seen for BA.1, BA.2, and BA2.75.2 (Fig. 1e). Early Clade Spike IgG antibody titers were also lower after Early Clade infection compared to those after vaccination in children (*p*=0.0006) though this was not statistically significant in adults (Fig. 1e, f). As expected, levels of Early Clade Spike IgG antibody in vaccinated children and adults, who received the Early Clade–based BNT162b2 vaccine, were high although not statistically different between age groups (Fig. 1f). As previously reported, titers of Early Clade Spike IgG antibody in ChAdOx1-vaccinated adults were lower than BNT162b2-vaccinated children and adults (*p*=0.0002 and *p*=0.002, respectively; Fig. 1f) [45]. Delta and Omicron infections tended to generate slightly higher titers than Early Clade infection across VOC Spikes without being affected by a more severe COVID-19 (Table 1; Fig. 1e, ns). Sera from Delta-infected individuals had 3- and 1.8-fold higher levels of Spike IgG when tested on Early Clade Spike in children and adults compared to titers against their cognate Delta Spike antigen without being statistically significant (Fig. 1e, ns). This trend was also observed in Omicron-infected children, who had 11-, 8-, 5-, 8-, 8-, and 11-fold higher levels of cross-reactive Early Clade Spike IgG compared to BA.1, BA.2, BA.5, BQ.1.1, BA2.75.2, and XBB.1 Spike IgG, respectively (Fig. 1e). Similarly, Omicron-infected adults had 6-, 4-, 3-, 5-, 4-, and 9-fold higher levels of Early Clade Spike IgG compared to BA.1, BA.2, BA.5, BQ.1.1, BA2.75.2, and XBB.1 Spike IgG, respectively (Fig. 1e). When children and adults were compared within the infected groups, pediatric antibody titers were similar to the ones induced in adults in Early Clade–, Delta-, and Omicron-infected groups, except for BA.2 in Delta-infected individuals (*p*=0.0224; Supplementary Table S2). In vaccinated individuals, there were significant differences between children and adults for all variants tested, except for Early Clade and Delta Spikes after immunization by BNT162b2 (Fig. 1f; Supplementary Table S2), and Delta Spike after ChAdOx1 (Supplementary Table S2).

### Strong Immunogenicity Is Induced Upon Natural Infection by Delta But Not by Omicron Variants

To determine whether infection by a specific VOC or Early Clade–based vaccination would confer an enhanced protection to emerging variants, we compared the antibody cross-reactivity across Spike VOCs developed by naturally infected and vaccinated individuals (Fig. 2a). The WHO International NIBSC Early Clade SARS-CoV-2 standard was serially diluted to assess the effect of concentration on Spike cross-reactive binding. The NIBSC standard SARS-CoV-2 antibody had limited to moderate binding toward Gamma and Alpha, followed by Beta, Delta, and Omicron BA.5, BA.2, BA.1 BQ.1.1, BA2.75.2, and XBB.1 Spikes (Fig. 2b, c). Importantly, the percentage of cross-reactive binding against all Spikes remained stable regardless of increasing antibody concentrations (Fig. 2b, c), enabling comparison between individuals with different titers of Spike IgG antibody.

Antibodies generated from natural infection by the Early Clade SARS-CoV-2 in children and adults had reduced binding toward all VOCs, moderate binding to Alpha and Gamma Spikes (50–75%, ns), weak to limited binding toward Beta Spike (around 40% in children and 25% in adults), weak binding to Delta Spike, and limited binding toward all Omicron subvariants BA.1, BA.2, BQ.1.1, BA2.75.2, and XBB.1 (10–20%), and BA.5 (median 25%) Spikes (Fig. 2d). This pattern of binding across VOCs was similar to that observed in the SARS-CoV-2 WHO standard, which is consistent with the use of pooled Early Clade–infected convalescent donors in this product (Fig. 2b, c). Delta-infected patient sera demonstrated strong binding toward Alpha, Delta, and Gamma Spike (>75%) in children and toward Alpha and Delta Spike in adults (>75%), with moderate binding (50–75%) toward Beta Spike in children and Beta and Gamma Spikes in adults. All sera had limited (<25%) binding toward Omicron BA.1, BA.2, BA2.75.2, and XBB.1, and weak binding to BA.5 and BQ.1.1 Spikes (Fig. 2e). Omicron-infected individuals had limited cross-reactive binding toward all Omicron subvariant Spikes, except for BA.5 to which the binding was weak (Fig. 2f). This was observed despite titers of BA.1, BA.2, BA.5, BQ.1.1, BA2.75.2, and XBB.1 Spike IgG being three- to five-fold higher compared to the ones in Early Clade– or Delta-infected individuals (Fig. 1e), suggesting low overall cross-reactive binding of these VOCs (Fig. 2d–f). Interestingly, patients naturally infected by Delta and Omicron had overall stronger binding across Alpha, Beta, and Gamma VOCs than individuals infected with the Early Clade SARS-CoV-2. Overall, there were no significant differences between adults and children in the binding profile across VOCs in all patients naturally infected (Fig. 2h, i, ns). Of all the naturally infected cohorts, the Delta-infected cohort displayed a robust cross-reactive binding toward all Spikes, including the cognate Delta Spike (Fig 2h, i, *p*<0.0001).

An infection-naïve vaccinated cohort of 24 individuals was also assessed (Table 1; Fig. 1a). BNT162b2- and ChAdOx1-vaccinated individuals showed a similar pattern of cross-reactive binding to VOCs as Early Clade–infected patients (Fig. 2d, g). Vaccinated individuals had moderate binding toward Alpha, Beta, and Gamma (50–75%), weak binding toward Delta (25–50%), BA.2, and BA.5 (around 35%), and limited binding toward BA.1, BQ.1.1, BA2.75.2, and XBB.1 Spikes (<25%) (Fig. 2g). Cross-reactive binding toward VOCs between individuals vaccinated with BNT162b2 and ChAdOx1 did not differ and was similar despite higher titers generated by the BNT162b2 vaccine in both children and adults (Fig. 1e; Fig. 2g). Moreover, there was no difference in the cross-reactive binding profile toward VOCs between infection-naïve vaccinated children and adults (Fig. 2h, i, ns).

### Specific Immunoreactive Regions Within Spike RBD Modulate Cross-Reactive Binding in Naturally Infected and Vaccinated Individuals

Given the strong immunoreactivity conferred by Delta, we compared the cross-reactive binding across the Epsilon, Kappa, and Delta Spikes, all bearing the immune-evasive mutation 452R (Fig. 3a). Notably, Kappa and Delta both originated from the same ancestral variant, but differ in the 484Q mutation found in Kappa Spike, and the 478K mutation found in Delta Spike. The WHO International NIBSC SARS-CoV-2 standard displayed weak binding toward Kappa Spike (25–50%), and limited binding toward Epsilon Spike (<25%). As previously seen, cross-reactive binding, expressed as percentage of binding compared to the Early Clade Spike, remained independent of increasing antibody concentrations (Fig. 3b). Sera from Early Clade–infected individuals exhibited weak binding toward Epsilon and Delta Spike (25–50%) and moderate binding toward Kappa Spike (50–75%). The 452R mutation within the RBD caused significant loss of immunoreactivity, and the 484Q mutation present in Kappa contributed to improved antibody binding (children *p*=0.0016; adults ns; Fig. 3c), while the 478K mutation present in the more recent Delta did not decrease antibody binding to a greater extent (Fig. 3c). All these individuals exhibited largely similar binding profiles toward Epsilon and Delta Spikes (Fig. 3c); therefore, subsequent evaluation of immunoreactivity was hence restricted to Kappa and Delta Spikes. Strikingly, although the addition of 478K was not beneficial to increase immunoreactivity to the same extent to 484Q in Kappa, sera from Delta-infected children and adults had a much stronger binding to all 452R-based Spikes with strong (75–100%) binding toward Delta Spike and even better binding to Kappa Spike (Fig. 3d). On the other hand, individuals naturally infected with Omicron SARS-CoV-2 demonstrated moderate (50–75%) binding toward Kappa Spike and comparatively lower (25–50%) binding toward Delta Spike (Fig. 3e). The extent of antibody binding toward Kappa and Delta Spike ranged from weak to moderate in the vaccinated cohorts as well, with children exhibiting better cross-reactive binding toward Kappa Spike than adults, regardless of the type of vaccine received (*p*<0.0001; Fig. 3f, g; Supplementary Table S2). Overall, the presence of the 478K mutation in Delta Spike and 484Q mutation in Kappa Spike imparted improved antibody binding, which translated into higher immunoreactivity observed in the cohort of Delta-infected individuals over other cohorts in consideration (*p*<0.0001; Fig. 3g), and could suggest that introduction of 484Q mutation would be beneficial for enhanced immunoreactivity in future vaccines.

Further evidence that the aa at position 484 is important for cross-reactive binding could also be seen when we analyzed binding to Eta, Gamma, and Beta, in which lysine (K) at position 484 has replaced glutamic acid (Q) present in Kappa (Fig. 3h). Early Clade–infected individuals displayed very strong binding toward Eta Spike (>90%) (Fig. 3j), and this very strong cross-reactive binding was observed in all naturally infected as well as vaccinated individuals regardless of their age groups (Fig. 3j–m). The presence of 501Y alone in Alpha led to a moderate overall cross-reactive binding (50–75%) (Fig. 3j). Unexpectedly, the beneficial effect of 484K was not demonstrated when in combination with 501Y in Beta and Gamma, and led to a reduction in binding toward Beta and Gamma Spikes in naturally infected individuals (Fig. 3j–l). Furthermore, the 417N mutation contributed to the decrease of antibody binding to Beta Spike, which was also observed to a lesser extent in 417T-bearing Gamma (Fig. 3j). This binding profile was observed in all cohorts tested (Fig. 3n).

### RBD Mutations in Omicron Spike Cause Significant Loss of Antibody Cross-Reactive Binding that Cannot be Fully Compensated by Epistasis

Antibody cross-reactive binding toward Omicron BA.1, BA.2, BA.5, BQ.1.1, BA2.75.2, and XBB.1 Spikes was significantly reduced in all naturally infected and vaccinated cohorts (Fig. 2). The 417N and 501Y mutations shown to play a role in reducing cross-reactive binding and present in preceding VOCs, such as Beta Spike, are also present in BA.1, BA.2, BA.5, BQ.1.1, BA2.75.2, and XBB.1 Spikes. In addition, all Omicron subvariant Spikes contain the 477N mutation and include many substitutions within the RBD and Spike (Fig. 4a). Given that the impact of mutations within the RBD can shift by epistasis, we assessed the binding to Spike in the naturally infected and vaccinated cohorts using naturally occurring 477N-bearing D.2 Spike, Omicron subvariant Spikes, and artificial AM1 and AM2 Spikes (Fig. 4a). As previously observed [12], 477N alone, common to all Omicron subvariant Spikes, induced a weak binding toward D.2 Spike across all cohorts (Fig. 4b–f). The addition of 484K and 501Y (common to Beta) further decreased the binding toward AM1 Spike, an artificial triple-mutated Spike (Fig. 4b–f). This suggests an important loss of immunoreactivity (>50%) directly caused by the 477N mutation which was compounded by 484K and 501Y, confirming the deleterious effect of this pair of mutations. However, this decrease in binding was compensated by 498R, a well-known epistatic contact mutation of 501Y, as evidenced by the strong binding observed when all sera were tested on AM2 Spike (Fig. 4b–f), an artificial Spike with three mutations at 477N, 498R, and 501Y (Fig. 4a). This binding profile was observed in all cohorts tested and was similar between children and adults (Fig. 4f, ns). When children and adults were compared within the vaccinated groups, there were no significant differences between age groups, except for AM1 Spike after immunization by BNT162b2 (Fig. 4e; Supplementary Table S2), and AM1, D2, and AM2 Spikes after ChAdOx1 (Fig. 4e; Supplementary Table S2).

## Discussion

The current study examines the humoral immune response and cross-reactive binding to Spike in COVID-19-naïve discrete groups of COVID-19 convalescent and vaccinated children and adults. Children and adults infected by SARS-CoV-2 displayed similar breadth and longevity of antibody responses generated against VOCs. Large overestimation of titers generated after infection by Delta and Omicron occurred when samples were not tested against their cognate Spike antigen. Vaccinated individuals of all ages, regardless of the type of vaccine, i.e., BNT162b2 or ChAdOx1, displayed a similar immunoreactivity profile to naturally infected individuals across all Spike variants analyzed. Notably, infection with Delta led to an enhanced cross-reactive binding that could not be readily explained by mutations within the RBD. Omicron infection, although able to induce higher antibody levels compared to infection with other variants, systematically resulted in a low cross-reactive binding. At the molecular level, a plausible explanation would be that the increase in binding due to epistatic interactions may be dampened by a large number of other immune-evasive mutations. This suggests that antibody breadth against the heavily mutated Omicron variants may be limited over the course of the pandemic. Our results reveal important molecular features central to the generation of high antibody titers and broad cross-reactive binding that should be considered in the development of future variant-proof vaccine design and global serosurveillance.

Studies comparing the humoral response upon SARS-CoV-2 infection between children and adults have led to various results with suggestion of a weaker humoral immune response and reduced antibody breadth in children in some [6, 10, 46–48], whereas recent studies have shown that children IgG responses stabilized better and were maintained at higher level than adults over time [9, 49]. In the current study, we observed that in inter-related children and adults, humoral responses and their longevity were robust and similar between all age groups. Furthermore, the breadth of recognition toward Spike VOCs and region of immunoreactivity within Spike RBD did not differ significantly across age groups, despite children having likely been exposed to other coronaviruses that could contribute to enhanced breadth of binding [50]. Similar to other studies [6, 9], our data show that humoral immunity generated from SARS-CoV-2 appears not to be compromised in children. This suggests that age-related differences in immunity may not be accounted by humoral responses, and alternatively, rapid elimination of the virus in children may be due to other causes, including a higher pre-activated innate immune system [51–53].

Our results highlight the importance of assessing Spike antibodies against the cognate antigen. While titers measured on Early Clade Spike could be very high, the ones obtained after measurement on Omicron Spikes were strikingly decreased in all cohorts, including in naturally Omicron-infected individuals. This is consistent with the structural analysis of both BA.1 and BA.2 Spike engaging in a structural conformation that is of lower antigenicity [54]. Given that many serological assays utilize an Early Clade Spike, this may lead to overestimation of the protection against breakthrough infection after immunization or infection. Indeed, the effectiveness of previous infection in preventing reinfection was estimated to be lower against Omicron compared to other VOCs, such as Beta and Delta [55], and breakthrough infections in vaccinated individuals have been widely reported [56]. Overall, low titers to Omicron variants observed across all groups could contribute to this population-based result.

SARS-CoV-2 virus accumulates mutations along the Spike protein, particularly within the primary antigenic RBD, to evade adaptive immunity, increase viral fitness, and in turn modulate RBD-affinity for ACE2 receptor and viral infectivity [57, 58]. Small changes in the right regions of the Spike glycoprotein can have significant influence on Spike tertiary and quaternary structures, and therefore on antibody binding. Indeed, although the Spike sequences of Delta [22, 57, 59, 60] and Kappa are almost identical, the change of E484Q in Kappa results in a Spike trimer configuration [61] that diverges from Delta and leads to increased cross-reactive binding. Our data also showed that individuals infected by the more immunogenic Delta exhibited better protection toward other VOCs compared to individuals infected with an earlier clade SARS-CoV-2. This could not be readily explained by mutations at immune-evasive positions 452 and 478 within the RBD. VOCs, like Delta, exhibit increased viral fitness as a result of furin cleavage at P681R which may increase cross-reactive binding at two levels, one being an increased viral load, and the second, conformational changes as a consequence of the cleavage of the S1 and S2 domains [62]. While this study focuses on antibody responses influenced by mutations within the RBD, non-RBD-binding antibodies may also contribute.

Our results suggest that although cross-reactive binding is observed after Omicron infection, antibodies generated by natural infection and vaccination across all ages bound weakly to Omicron subvariants. Omicron variants are widely recognized as highly transmissible SARS-CoV-2 variants with evidence of immune escape and high capacity to evade antibody-mediated neutralization [25, 59, 63, 64]. The limited binding toward Omicron subvariant Spikes may explain their high capacity of immune evasion and high transmissibility, and hence higher infectivity rate and recurrent infection in the era of high proportions of convalescent and/or vaccinated communities. The Omicron subvariants differ by a small number of changes, and their global emergence may be explained by immune-evasive substitutions at positions 452 (from BA.5) and 486. The immune evasion is even more evident in XBB.1, BQ1.1, and BA.2.75.2 which have acquired a mutation at aa 346 [65, 66]. Overall, the low cross-reactive binding to all Omicron subvariants is an important factor to consider for pandemic control and vaccine design, especially in the context of the limited number of vaccine boosters and low uptake of bivalent vaccines available to the pediatric population worldwide.

We showed that the 501Y mutation significantly reduced antibody binding in most VOCs and VOIs and in all age groups, even on its own as in Alpha. 501Y had an additive effect to substitutions at positions 417 and 484 as seen in the Beta and Gamma VOCs. Strong decrease in binding was also observed when 477N, a deleterious mutation, was combined to 501Y in an artificial Spike (AM1 Spike) and promoted antibody escape. However, 484K on its own, as in Eta, despite having been reported as a class 2 escape mutation for neutralization of monoclonal antibodies [67], did not induce strong decrease of antibody binding. A similar trend was also observed in 484Q together with 452R (Kappa). Specific mutations can potentiate others at different sites, which can, in turn, influence the binding, affinity, or function of antibodies, a phenomenon known as epistasis [68, 69]. We provided evidence for the first time that epistasis occurs across natural infection and vaccination in children and adults. An obvious epistatic effect was observed in another artificial Spike (AM2 Spike) when 498Q was introduced in conjunction to 501Y and led to no loss of cross-reactive binding despite the presence of 477N. This combination of 501Y and 498Q in Omicron stabilizes the RBD conformation through salt bridge with a higher affinity to ACE2 [69, 70], and simultaneously promotes antibody accessibility and increases cross-reactive binding. Importantly, this was observed across all age groups and cohorts and infection with multiple variants, suggesting a conserved mechanism that could assist the breadth of immunoprotective antibodies.

COVID-19 vaccines have shown great success and have been proven effective at protecting against SARS-CoV-2 reinfection and reducing severe clinical outcomes [71]. A protective antibody response depends on antibody concentration and cross-reactive binding. We show that although second dose mRNA-based vaccination does indeed generate high Spike antibody titers in children and adults, the ability of these antibodies to bind to emerging Spike variants and subsequently neutralize the virus varies considerably. Overall, although there were differences in titers, both types of vaccines led to similar antibody cross-reactive binding. Based on our results, high Spike antibody titers generated after immunization coupled with mutations that generate strong cross-reactive binding, such as in Delta, are factors for consideration in future vaccine design. This could potentially be reinforced by harnessing the beneficial effects of combined mutations to enhance antibody binding and combat emerging variants. Interestingly, several vaccine boosters have now been developed and been based on an Omicron sequence and preliminary data show increased neutralization to Omicron VOC [72].

This study has a number of limitations. Firstly, although naturally infected individuals in our cohorts belonged to same households, were inter-related, and therefore had increased probability to be infected by the same variant, they still represented a relatively small sample size, and generalizability of the humoral immune responses needs to be carefully interpreted. Severity of COVID-19 was mostly mild or asymptomatic in naturally infected individuals, and viral loads were unknown. Although all individuals were COVID-19-naïve at recruitment, some participants may have suffered an asymptomatic reinfection generating very low titers of Spike antibody during the course of follow-up sampling. Serum antibodies have been studied and binding to Spike has been assessed by flow cytometry. Antibody binding to a trimeric/conformational Spike, as herein, might be a complementary approach to determining its role in in vivo protection against disease severity. For instance, sotrovimab poorly neutralizes live BA.2 in vitro, but can readily bind its Spike which is a better predictor of reducing disease than neutralization capacity in animal models [54, 73]. Spike binding and live virus neutralization are well correlated for the Early Clade viruses [12], and less so for Omicron subvariants for which binding can exceed neutralization [27, 28]**.** Finally, as it is often the case with studies on children, small volumes of blood were collected from participants and further studies on peripheral cells could not be performed.

Global control of the COVID-19 pandemic requires therapeutic and evidence-based adaptation to the rapidly evolving SARS-CoV-2 virus. The current study places emphasis on the importance of regions of antibody immunoreactivity on Spike to understand the antibody response mounted upon infection and vaccination. Results from this study will ultimately aid strategies of vaccine development to initiate robust and broadly protective adaptive immune responses.

### Supplementary Information

ESM 1: Supplementary Table 1. List of Spike variants and mutations of interest. Supplementary Table 2. Comparison of Spike antibody responses in children versus adults. Supplementary Figure 1: Correlation between flow cytometry Spike antibody and live neutralization assays. Spike IgG titers were correlated with live virus neutralization of Early Clade (a), Delta (b), BA.1 (c), BA.2 (d), BA.5 (e), BQ.1.1 (f), and XBB.1 SARS-CoV-2 (g). R^2^ and p values after linear regression are shown. ΔMFI = delta median fluorescence intensity; Ab = antibody. Supplementary Figure 2: Persistence of serum Spike antibodies against other natural variants of interest (VOI) in children and adults. (a) Schematic indicating mutations in VOIs within the Spike protein. D614 IgG responses against (b) B clade 20Fa, (c) Zeta, (d) Epsilon, (e) A.23.1/Uganda, (f) Eta, (g) Iota and (h) Kappa Spike VOIs over time in Early Clade-infected children (ages 0-18 years old, peach) and adults (>18 years old, grey) shown since viral exposure. (DOCX 490 KB)

## Data Availability

Any data within the article will be shared in anonymized format by request from qualified investigators. If desired, please contact the corresponding author of this article.
