# Supplementary material for: Infection and Vaccine Induced Spike Antibody Responses Against SARS-CoV-2 Variants of Concern in COVID-19-Naïve Children and Adults
Source: J Clin Immunol. 2023 Jul 5;43(8):1706–23. doi: 10.1007/s10875-023-01540-5 (PMC10661752; doi:10.1007/s10875-023-01540-5)
Supplement: Supplementary file 1 — Supplementary Table 1. List of Spike variants and mutations of interest. Supplementary Table 2. Comparison of Spike antibody responses in children versus adults. Supplementary Figure 1: Correlation between flow cytometry Spike antibody and live neutralization assays. Spike IgG titers were correlated with live virus neutralization of Early Clade (a), Delta (b), BA.1 (c), BA.2 (d), BA.5 (e), BQ.1.1 (f), and XBB.1 SARS-CoV-2 (g). R2 and p values after linear regression are shown. ΔMFI = delta median fluorescence intensity; Ab = antibody. Supplementary Figure 2: Persistence of serum Spike antibodies against other natural variants of interest (VOI) in children and adults. (a) Schematic indicating mutations in VOIs within the Spike protein. D614 IgG responses against (b) B clade 20Fa, (c) Zeta, (d) Epsilon, (e) A.23.1/Uganda, (f) Eta, (g) Iota and (h) Kappa Spike VOIs over time in Early Clade-infected children (ages 0-18 years old, peach) and adults (>18 years old, grey) shown since viral exposure. (DOCX 490 KB) [file 10875_2023_1540_MOESM1_ESM.docx]

**Infection and vaccine induced Spike antibody responses against SARS-CoV-2 Variants of Concern in COVID-19 naïve children and adults**

Aleha Pillay^a,b^*, Avani Yeola^a^*, Fiona Tea^a^, Martina Denkova^a^, Samuel Houston^a^, Rebecca Burrell^b,c^, Vera Merheb^a^, Fiona X. Z. Lee^a^, Joseph A. Lopez ^a^, Lilly Moran^c,d^, Ajay Jadhav^c,d^, Katrina Sterling^c,d^, Catherine L. Lai^c^, Tennille L. Vitagliano^c^, Anupriya Aggarwal^e^, Dan Catchpoole^c^, Nicholas Wood^c,d^, Tri Giang Phan^f,g^, Ralph Nanan^h^, Peter Hsu^c,i^, Stuart G. Turville^e^, Philip N. Britton^c,j^, and Fabienne Brilot^a, b,j,k^.

**Affiliations**

1. Brain Autoimmunity Group, Kids Neuroscience Centre, Kids Research at the Children’s Hospital at Westmead, Sydney, New South Wales, Australia
2. School of Medical Sciences, Faculty of Medicine and Health, The University of Sydney, Sydney, New South Wales, Australia
3. Kids Research at the Children’s Hospital at Westmead, Sydney, New South Wales, Australia
4. National Center for Immunisation Research and Surveillance, the Sydney Childrens Hospitals Network, Sydney, New South Wales, Australia
5. The Kirby Institute, The University of New South Wales, Sydney, New South Wales, Australia
6. Garvan Institute of Medical Research, Sydney NSW, Australia
7. St Vincent’s Healthcare Clinical Campus, School of Clinical Medicine, Faculty of Medicine and Health, The University of New South Wales, Sydney, New South Wales, Australia
8. Charles Perkins Center and Sydney Medical School Nepean, Faculty of Medicine and Health, The University of Sydney, Sydney, New South Wales, Australia
9. Department of Allergy and Immunology, The Children’s Hospital at Westmead, New South Wales, Australia
10. Sydney Institute for Infectious Disease, Faculty of Medicine and Health, The University of Sydney, Sydney, New South Wales, Australia
11. Brain and Mind Centre, The University of Sydney, Sydney, New South Wales, Australia

* Contributed equally to this work.

**Correspondence:**

Fabienne Brilot, Brain Autoimmunity Group, Kids Neuroscience Centre, Kids Research at the Children’s Hospital at Westmead, School of Medical Sciences, Faculty of Medicine and Health, The University of Sydney, Sydney, New South Wales, Australia, Fabienne.brilot@sydney.edu.au

**Journal of Clinical Immunology**

**Supplemental Material**

**Table of content**

Supplementary Methods (Page 2-3)

Supplementary Table 1 (Page 3-4)

Supplementary Table 2 (Page 4-6)

Supplementary Figure 1 (Page 7)

Supplementary Figure 2 (Page 8)

Supplementary Figure legends (Page 8)

**Supplementary Methods**

## Detection of Spike antibody immunoreactivity by live flow cytometry cell-based assay

## For each variant analysed, serum (1:80) was added to live Spike-transfected cells, followed by AlexaFluor 647-conjugated anti-human IgG (H+L), IgA, or IgM (ThermoFisher Scientific) as previously reported. All Spike constructs were cloned into the same bicistronic vector permitting simultaneous expression of the intracellular ZsGreen reporter molecule and cell surface Spike. ZsGreen expression was used to normalize for Spike expression to allow comparisons between variants. Four patient sera of known Spike IgG titer were run concurrently as controls. Cells were acquired on the LSRII flow cytometer through a HTS (BD Biosciences). Patients were determined Spike antibody-seropositive if the Delta median fluorescence intensity (ΔMFI = MFI transfected cells – MFI untransfected cells) was above the positive threshold (mean ΔMFI+4SD of 24 age-matched pre-pandemic controls) in at least two of three quality-controlled experiments. Paediatric and adult control sera background fluorescence and positive thresholds were similar (data not shown), therefore paediatric controls were used throughout to assess Spike seropositivity. Data was analysed using FlowJo 10.4.1 (TreeStar, USA), Excel (Microsoft, USA), and GraphPad Prism (GraphPad Software, USA).

**Correlation between live flow cytometry cell-based assay and live virus neutralization assay by rapid high-content SARS-CoV-2**

Rapid high-content live virus neutralization assay was performed as previously described (26, 28, 65). Briefly, anti-SARS-CoV-2 hyperimmune globulins (Privigen 538) were serially diluted in DMEM-5% FBS and mixed in duplicate with an equal volume of SARS-CoV-2 virus solution standardized at 2× median Virus Effective (VE_50_). After 1 h of virus–antibody coincubation at 37 °C, 40 μL were added to an equal volume of nuclear-stained HAT-24 cells pre-plated in 384-well plates. Plates were incubated for 20 h before enumerating nuclear counts with a IN Cell Analyzer 2500HS high-content microscope and IN Carta analysis software (Cytiva, USA). The % neutralization was calculated with the formula: Neutralization (%) = (D − (1 − Q)) × 100/D as previously described (12). “Q” is a well's nuclei count divided by the average count for uninfected controls (defined as having 100% neutralization) and D = 1 − Q for the average count of positive infection controls (defined as having 0% neutralization). Sigmoidal dose–response curves were obtained with GraphPad Prism software. Spike antibody titers (IgG) were obtained by live flow cytometry cell-based assay with serial 1:3 dilutions anti-SARS-CoV-2 hyperimmune globulins (Privigen 538) ranging from 9 and 0.0004 UI/ml. Spike antibody titers were interpolated from a non-linear 4-parameter logistic (4PL) regression model to obtain titers that corresponded to concentrations used in the live virus neutralization assay (GraphPad Prism software). Spike antibody titers and neutralization percentages were correlated after linear regression in GraphPad Prism software (shown in Supplementary Fig. S1).

**Statistics**

Non parametric Kruskal-Wallis test followed by Dunn’s multiple comparison test were performed and adjusted p values were shown in figures when significant and highlighted differences between cross-reactive binding towards VOC Spikes versus Early Clade Spike. Two-way ANOVA with main effect-only model (GraphPad v9.4.1) was also applied to compare overall cross-reactive binding profiles between paediatric and adult cohorts (Fig. 2h, i; Fig. 3g, n; Fig. 4f) and p values were shown in the text. Correlation between Spike IgG titres and live neutralization was assessed by linear regression, and the coefficient of determination (R^2^) and p values were reported. Power calculations based on the hypothesis of 42% difference (effect size) between Spike antibody titers in Early Clade-infected children and adults (assuming alpha value of 0.05 and 80% power, and a ratio of children to adults of 0.25) led to a required sample size of n=9 children and n=2 adults. Given these parameters, our sample size of n=14 children and n=4 adults would have a power of 99.9% assuming a standard deviation of 20% (early clade infected at the immune response peak) to detect the hypothesised effect size. Our results showed a 5% decrease in Spike antibody titers from children to adults (instead of 42%). %). For this difference to be detected as statistically significant, the study would require a recruitment of n = 628 children and n = 157 adults. Our study is underpowered to show a difference of 5% in the Spike Ab titers between children vs adults.

**Supplementary Table 1. List of Spike variants and mutations of interest**

| **Variant type** | **Variant number** | **Variant name** | **Pango lineage** | **Mutations of interest^a^** | **Deletions/**  **Insertions** |
| --- | --- | --- | --- | --- | --- |
| VOC | 1 | D614 |  |  |  |
| VOC | 2 | D614G |  | D614G |  |
| VOC | 3 | Alpha | B.1.1.7 | **N501Y,** A570D, D614G, P681H, T716I, S982A, D1118H | H69/V70del, V143_Y145del |
| VOC | 4 | Beta | B.1.351 | D80A, D215G,  R246I,  **K417N, E484K, N501Y,** D614G, A701V | L242_L244del |
| VOC | 5 | Gamma | P.1 | L18F, T20N, P26S, D138Y, R190S, **K417T, E484K, N501Y,** D614G, H655Y, T1027I, V1176F |  |
| VOC | 6 | Delta | B.1.617.2 | T19R, G142D, R158G, **L452R, T478K,** D614G, P681R, D950N |  |
| VOC | 7 | Omicron BA.1 | B.1.1.529.1 | A67V, T95I, G142D, L212I, **G339D, S371L, S373P, S375F, K417N, N440K, G446S, S477N, T478K, E484A, Q493R, G496S, Q498R, N501Y, Y505H**, T547K, D614G, H655Y, N679K, P681H N764K, D796Y, N856K, Q954H, N969K, L981F | H69_V70del, V143_Y145del, N211del, R214_insEPE, |
| VOC | 8 | Omicron BA.2 | B.1.1.529.2 | T19I, A27S, G142D, V213G, **G339D, S371F, S373P, S375F, T376A, D405N, R408S, K417N, N440K, S477N, T478K, E484A, Q493R, Q498R, N501Y, Y505H,** D614G, H655Y, N679K, P681H, N764K, D796Y, Q954H, N969K | PPA24_26del |
| V | 9 | BA.2.75.2 |  | T19I, A27S, G142D, K147E, W152R, F157L, I210V, V213G, G257S, **G339H, R346T, S371F, S373P, S375F, T376A, D405N, R408S, K417N, N440K, G446S, N460K, S477N, T478K, E484A, F486S, ~~Q493R,~~ Q498R, N501Y, Y505H,** D614G, H655Y, N679K, P681H, N764K, D796Y, Q954H, N969K, D1199N | PPA24_26del |
| VOC | 10 | XBB.1 |  | T19I, A27S, V83A, G142D, H146Q, Q183E, V213E, **G339H, R346T, L368I, S371F, S373P, S375F, T376A, D405N, R408S, K417N, N440K, V445P, G446S, N460K, S477N, T478K, E484A, F486S, F490S, Q498R, N501Y, Y505H,** D614G, H655Y, N679K, P681H, N764K, D796Y, Q954H, N969K | PPA24_26del  144del |
| VOC | 11 | Omicron BA.5 | B.1.1.529.5 | T19I, A27S, G142D, V213G, **G339D, S371F, S373P, S375F, T376A, D405N, R408S, K417N, N440K, L452R, S477N, T478K, E484A, F486V, Q498R, N501Y, Y505H,** D614G, H655Y, N679K, P681H, N764K, D796Y, Q954H, N969K | PPA24_26del  H69_V70del |
| V | 12 | BQ.1.1 | B.1.1.529.5.3.1.1.1.1.1.1 | T19I, A27S, G142D, V213G, **G339D, R346T, S371F, S373P, S375F, T376A, D405N, R408S, K417N, N440K, K444T, L452R, N460K, S477N, T478K, E484A, F486V, Q498R, N501Y, Y505H,** D614G, H655Y, N679K, P681H, N764K, D796Y, Q954H, N969K | PPA24_26del  H69_V70del |
| VOI | 13 | Epsilon | B.1.429 | S13I, W152C, **L452R,** D614G, Q677H |  |
| VOI | 14 | Kappa | B.1.617.1 | G142D, E154K, **L452R, E484Q,** D614G, P681R, Q1071H |  |
| VOI | 15 | Iota | B.1.526 | L5F, T95I, D614G, D253G A701V, **E484K** |  |
| VOI | 16 | B clade 20F**^b^** | D.2 | S477N, D614G |  |

**a** Bold indicates mutation/s within the Receptor Binding Domain (RBD)

**b** No WHO nomenclature available for this variant.

**Supplementary Table 2. Comparison of Spike antibody responses in children versus adults.**

| **Comparison** | **Spike Variant** | **Comparison of Spike Ab titer ^a^** | **Comparison of Spike Ab cross-reactive binding ^a^** |
| --- | --- | --- | --- |
| Early Clade infected Children vs. Early Clade infected Adults | Early Clade | 5.8832 | N/A |
|  | Alpha | nd | 11.55 |
|  | Beta | nd | 1.845 |
|  | Gamma | nd | 3.699 |
|  | Delta | 1.8616 | 11.0895 |
|  | Kappa | nd | 4.713 |
|  | Eta | nd | 7.62 |
|  | AM1 | nd | 8.406 |
|  | D.2 | nd | 8.1225 |
|  | AM2 | nd | 14.9985 |
|  | BA.1 | 3.0536 | 12.3495 |
|  | BA.2 | 3.8184 | 12.0975 |
|  | BA.5 | 4.3896 | 1.818 |
|  | BQ.1.1 | 7.6504 | 0.7755 |
|  | BA.2.75 | 5.6272 | 0.8505 |
|  | XBB | 5.6272 | 1.1415 |
| Delta infected Children vs. Delta infected Adults | Early Clade | 0.5832 | N/A |
|  | Alpha | nd | 9.333 |
|  | Beta | nd | 11.688 |
|  | Gamma | nd | 3.1905 |
|  | Delta | 0.32 | 0.447 |
|  | Kappa | nd | 0.9765 |
|  | Eta | nd | 14.7045 |
|  | AM1 | nd | 2.8935 |
|  | D.2 | nd | 0.096 |
|  | AM2 | nd | 0.435 |
|  | BA.1 | 0.288 | 13.7415 |
|  | BA.2 | **0.0224** | 4.548 |
|  | BA.5 | 0.32 | 0.714 |
|  | BQ.1.1 | 0.4024 | 2.3025 |
|  | BA.2.75 | 0.252 | 2.706 |
|  | XBB | 0.252 | 2.094 |
| Omicron infected Children vs. Omicron infected Adults | Early Clade | 5.3336 | N/A |
|  | Alpha | nd | 0.357 |
|  | Beta | nd | 1.428 |
|  | Gamma | nd | 1.965 |
|  | Delta | 0.7616 | 13.572 |
|  | Kappa | nd | 10.7145 |
|  | Eta | nd | 2.8575 |
|  | AM1 | nd | 11.4285 |
|  | D.2 | nd | 6.072 |
|  | AM2 | nd | 2.8575 |
|  | BA.1 | 0.1904 | 8.214 |
|  | BA.2 | 2.2856 | 7.857 |
|  | BA.5 | 1.3336 | 2.1435 |
|  | BQ.1.1 | 1.3336 | 14.4645 |
|  | BA.2.75 | 0.3808 | 2.5005 |
|  | XBB | 1.3336 | 2.1435 |
| Vaccinated Children vs. BNT Vaccinated Adults | Early Clade | 0.6232 | N/A |
|  | Alpha | nd | 11.6685 |
|  | Beta | nd | 3.9975 |
|  | Gamma | nd | 13.4655 |
|  | Delta | 0.3032 | 14.0745 |
|  | Kappa | nd | 0.003 |
|  | Eta | nd | 12.846 |
|  | AM1 | nd | **0.003** |
|  | D.2 | nd | 4.599 |
|  | AM2 | nd | 5.52 |
|  | BA.1 | **0.0088** | 0.0045 |
|  | BA.2 | **0.0016** | 1.4565 |
|  | BA.5 | **0.0016** | 0.2055 |
|  | BQ.1.1 | **0.0016** | 2.9655 |
|  | BA.2.75 | **0.0016** | 0.081 |
|  | XBB | **0.0016** | 7.8315 |
| Vaccinated Children vs. ChAd Vaccinated Adults | Early Clade | **0.0016** | N/A |
|  | Alpha | nd | 14.0535 |
|  | Beta | nd | 1.629 |
|  | Gamma | nd | 11.718 |
|  | Delta | 0.3032 | 5.1075 |
|  | Kappa | nd | **0.003** |
|  | Eta | nd | 7.3215 |
|  | AM1 | nd | **0.003** |
|  | D.2 | nd | **0.003** |
|  | AM2 | nd | **0.012** |
|  | BA.1 | **0.0016** | 0.2865 |
|  | BA.2 | **0.0016** | 6.729 |
|  | BA.5 | **0.0016** | 2.7825 |
|  | BQ.1.1 | **0.0016** | 5.49 |
|  | BA.2.75 | **0.0016** | 3.927 |
|  | XBB | **0.0016** | 8.856 |

**a** Mann-Whitney U test with Bonferroni’s correction for multiple comparisons. Significant corrected P values are indicated in bold.

nd: not determined

N/A: not applicable


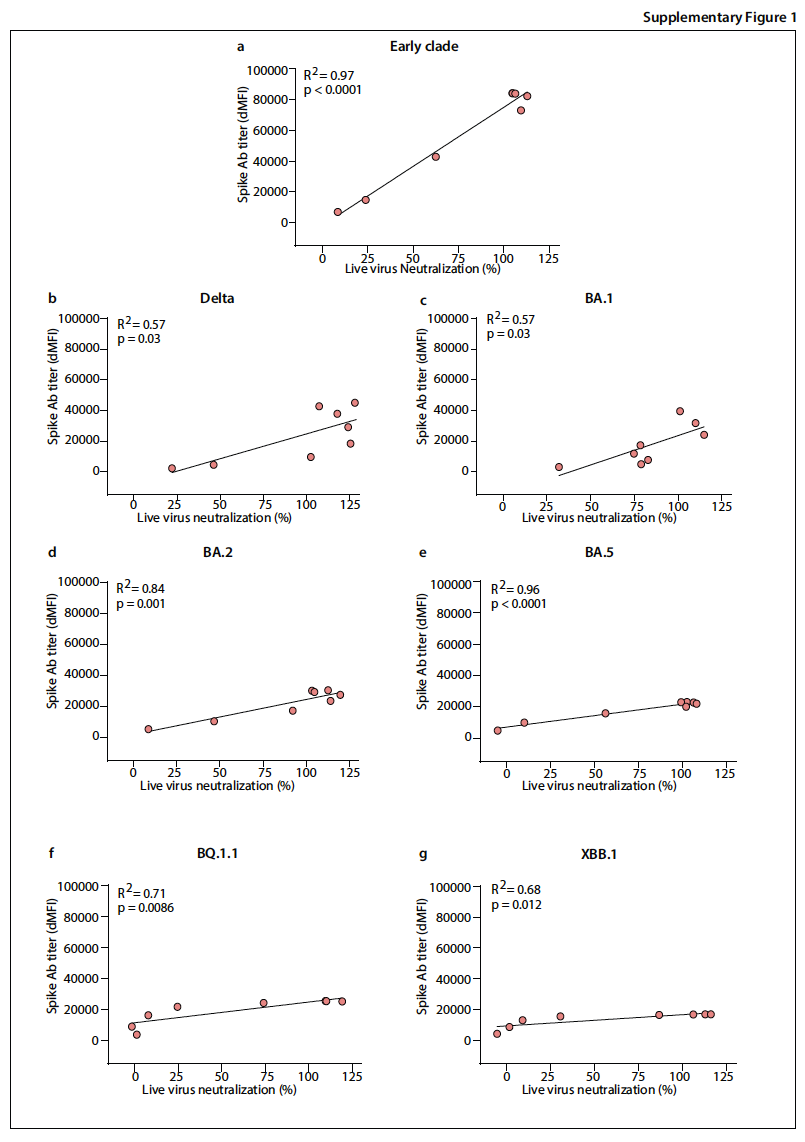


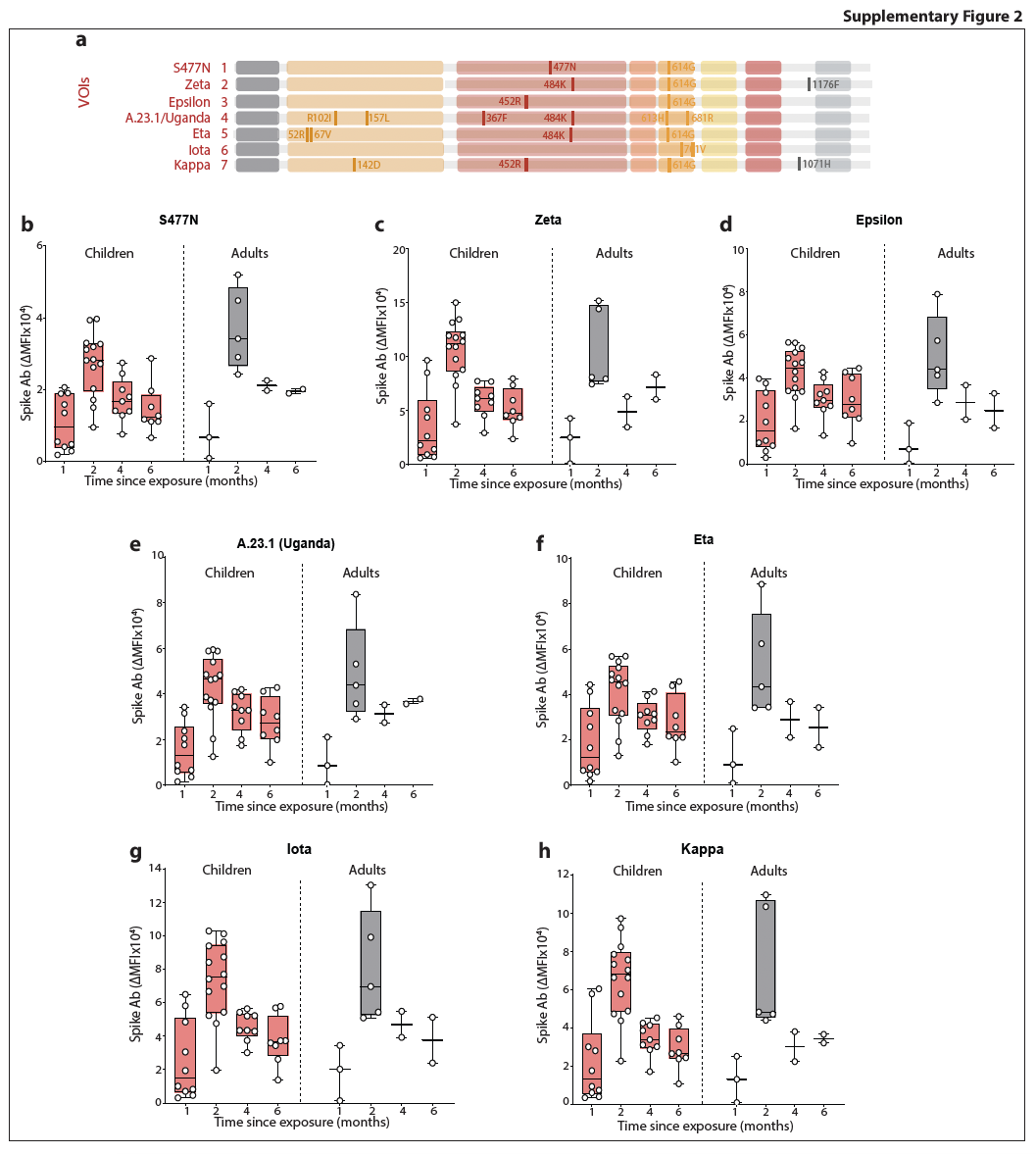


**Supplementary Figure legends**

**Supplementary Figure 1: Correlation between flow cytometry Spike antibody and live neutralization assays.** Spike IgG titers were correlated with live virus neutralization of Early Clade (**a**), Delta (**b**), BA.1 (**c**), BA.2 (**d**), BA.5 (**e**), BQ.1.1 (**f**), and XBB.1 SARS-CoV-2 (**g**). R^2^ and p values after linear regression are shown. ΔMFI=delta median fluorescence intensity**;** Ab = antibody

**Supplementary Figure 2: Persistence of serum Spike antibodies against other natural variants of interest in children and adults. (a)** Schematic indicating mutations in VOIs within the Spike protein. D614 IgG responses against (**b**) B clade 20Fa, (**c**) Zeta, (**d**) Epsilon, (**e**) A.23.1/Uganda, (**f**) Eta, (**g**) Iota and (**h**) Kappa Spike VOIs over time in Early Clade-infected children (ages 0-18 years old, peach) and adults (>18 years old, grey) shown since viral exposure.
